# Nanoporous carbons based on coordinate organic polymers as an efficient and eco-friendly nano-sorbent for adsorption of phenol from wastewater

**DOI:** 10.1038/s41598-023-40243-0

**Published:** 2023-08-12

**Authors:** Soheila Sharafinia, Alimorad Rashidi, Behnam Babaei, Yasin Orooji

**Affiliations:** 1 Department of Chemistry, Faculty of Science, Shahid Chamran University of Ahvaz, Ahvaz, Iran; 2 Nanotechnology Research Center, Research Institute of Petroleum Industry (RIPI), Tehran, Iran; 3 Department of Chemistry, Faculty of Basic Science, University of Mohaghegh Ardabili, Ardabil, Iran; 4 Material and Energy Research Center, Karaj, Alborz Iran

**Keywords:** Environmental sciences, Environmental social sciences, Chemistry, Nanoscience and technology

## Abstract

The major part of water pollutants includes of organic such as phenolic pollutant, thus there are every hazardous to environment. Present work is a comparative onto surface chemistry and adsorptive characteristics of coordinate organic polymer (Cop-150) and nanoporous carbon (NPC) prepared using solvothermal method. New NPC was successfully synthesized to remove of phenol. FT-IR, XRD, XPS, SEM, TGA, and BET techniques have been used to characterization and confirm physicochemical variation during preparing Cop-150 and NPC. Box–Behnken response surface methodology (BBRSM) was used to optimize four important factors of the pH (2–10), contact time (1–40 min), temperature (25–60 °C), and initial concentration of phenol (5–50 mg L^−1^). To analyze the data obtained from the adsorption of phenol by synthesized adsorbents, four linear, 2FI, quadratic and cubic models were examined, which the quadratic model was recognized as the best model. To the NPC the equal adsorption capacity 500 mg g^−1^ is achieved at the initial concentration of phenol = 49.252 mg L^−1^, contact time = 15.738 min, temperature = 28.3 °C, and pH 7.042. On the other hand, the adsorption capacity for Cop-150 in pH 4.638, the contact time = 19.695 min, the temperature = 56.8 °C, and the initial concentration of phenol = 6.902 mg L^−1^ was equal to 50 mg g^−1^. The experimental data at different conditions were investigated by some famous kinetic and isotherm models, which among them, were corresponded to the pseudo-second-order kinetic model and the Langmuir isotherm. Moreover, based to result of thermodynamics to the both Cop-150 and NPC, the adsorption process is exothermic and spontaneous. According to results the Cop-150 and NPC could be used for up to four and five cycles without significantly reducing their performance, respectively.

## Introduction

Water pollution happens with the entry of industrial chemicals into the water, which is associated with changes in water quality. These compounds are very harmful to the environment, and human life, and have negative effects on ecosystems. Phenol (see Table 1) is an industrial compound widely used in plastic and resin, paper, coal refineries, and petrochemical industries. This compound and its derivatives are very toxic, and are cause diseases, such as poisoning^1^. The increasing presence of phenol in wastewater has become a pressing concern due to its harmful effects on both human health and the environment^2^. Phenol is a negative effect on organisms even in very little amounts. Based on the World Health Organization, the concentration range of the phenolic compounds in drinking water is about 1 µg L^−1^, therefore, need to be removed from the water stream. To dissolve this problem, various strategies such as distillation, ion exchange^3^, membrane-based filtration, biochemical reduction, chemical oxidation/reduction, and adsorption have been investigated for wastewater treatment^4–7^. Among these technique, the adsorption process is most used in wastewater treatment due to its high economic efficiency, impressive capacity, and excellent performance^8–11^. In recent years, kinds of adsorbents such as metal oxides^12,13^, magnetic nanoparticles^6,14–16^, polymers^17^, and graphene-based materials^18^ have been studied to treatment of wastewater. But, the preparation of these materials is expensive and they have a very low surface area^19^, which as result reduced the efficiency of the adsorption process^20,21^. To combat this issue, researchers have been exploring various adsorbents to effectively remove phenol from wastewater. One promising solution lies in the use of NPCs based on coordinate organic polymers, which have shown great potential as efficient and eco-friendly nano-sorbents^22^. NPCs are very promising because of their unique structure, high porosity, and suitable surface for use in different research works, including drug delivery systems, supercapacitors, gas storage, and adsorption of pollutants^22^.

**Table 1:** The physiochemical properties of the phenol.

Molecular structure	
3D model structure	
Molecular formula	C_6_H_6_O_1_
Molecular weight (g mol^−1^)	94.11
λmax (nm)	269

The adsorption of phenol by NPC is a novel approach to addressing the removal of phenol from wastewater and other industrial processes. NPC materials offer a high surface area and unique pore structure, which makes them highly effective in adsorbing organic pollutants like phenol^23^. The NPCs structure provides a large number of active sites for phenol molecules to interact with, allowing for efficient removal from solution^24,25^. Furthermore, NPC offers excellent adsorption capacity and fast kinetics, allowing for rapid and efficient phenol removal. The high surface area and pore volume of NPC materials provide ample space for phenol molecules to be adsorbed, resulting in high removal efficiencies. Once the carbon material becomes saturated with phenol, it can be easily regenerated through processes such as thermal desorption or solvent extraction, allowing for multiple cycles of phenol removal. Overall, the adsorption of phenol by NPC presents a novel and promising approach to addressing the removal of phenol from various industrial processes^23^.

In addition to their efficiency, NPCs based on coordinate organic polymers offer eco-friendly benefits. The synthesis process of these nano-sorbents also minimizes the use of harsh chemicals and energy-intensive procedures, further contributing to their eco-friendly nature^23^.

Recently, to the production of microporous carbons, coordinate organic polymers (Cops) are very suitable and new candidates. These polymers are metal ions made in coordination with rigid organic molecules, which are used to forming one-, two-, or three-dimensional structures. Via choosing suitable Cops and MOFs with high thermal stability, the carbonization of carbon sources occurs inside the micropores, and the original porous structures of Cops and MOFs are thus retained. For example, using furfuryl alcohol as a carbon source, its molecular dimensions make it sufficient for entering and filling the framework of MOF-5 (zeolite-type MOF, ZIF-8) or Al-based Cop. After the carbonization process of this alcohol, the achieved NPCs had a larger surface area. Therefore, considering such promising effects, the preparation of carbons derived from MOF or Cop deserves more attention. Since MOFs and Cop contain a large amount of carbon content, the presence of additional carbon sources as additives (such as furfuryl alcohol) is not always necessary. This idea enables the motivation to examine a new method of direct conversion of MOFs or Cops. In this research work, we have selected the flexible Al-based Cop-150 with a carbon–carbon bonded and porous structure as an initial precursor^26^. These polymers have a suitable, scalable, flexible, and affordable synthesis. In the experiments, Cop-150 powders were synthesized as the initial precursor and then treated at a calcination temperature of 800 °C to increase the surface area. The NPC has shown high adsorption capacity to phenol molecules removal. The maximum capacity of phenol using NPC was 500 mg g^−1^, which is a significant amount compared to many adsorbents reported in other literature. In addition, Design-Expert software was also used to analyze the test results.

## Experimental

### Materials

All used chemical materials in this study, aluminum chloride anhydrous (AlCl_3_, 95%), absolute methanol (MeOH, 99.0%), absolute ethanol (EtOH, 99.0%), 1,2-dichloroethane (DCE, 99.0%), dichloromethane (DCM, 99.5%), chloroform (99.5%), benzene (99.5%), Hydrofluoric acid (HF, 48%) with high purity have been purchased from Merck (Darmstadt, Germany**).**

### Synthesis of Cop-150

Cop-150 was prepared by the solvothermal method according to the reported procedure in the previous literature^26^.

**Figure 1:**
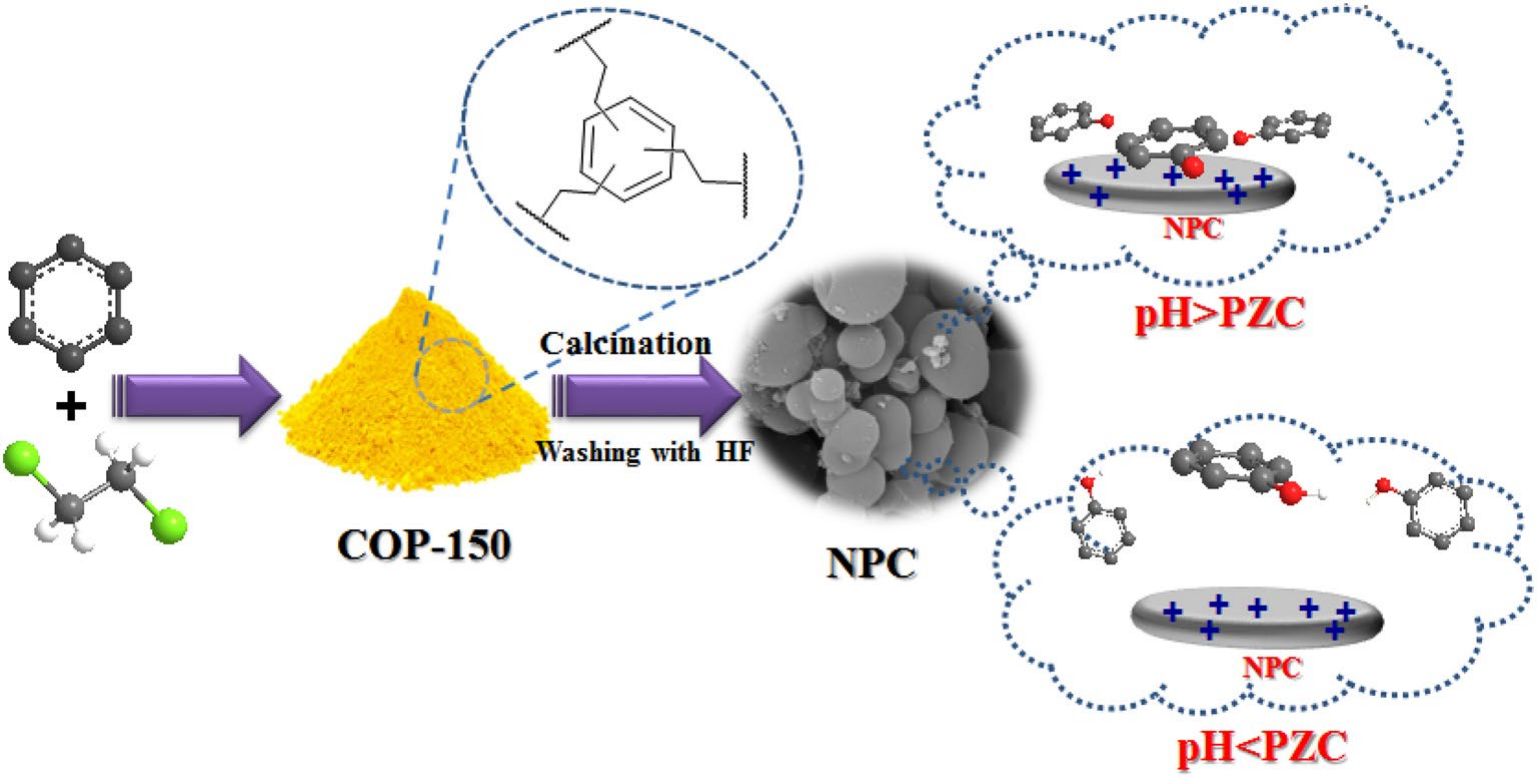
Synthesis of NPC and mechanism of phenol adsorption by NPC at positive and negative pHs.

In a 500 mL beaker, a solution consisting of 200 DCE and 10 mL of benzene was added and stirred for 5 min. Then 30 g of anhydrous AlCl_3_ was added to the resulting solution and stirred at room temperature for 1 h. Afterward 1 h, the stirring was stopped due to the accumulation of particles around the stirrer bar. After 24 h, by mechanically breaking pieces of aggregates and slowly adding 200 mL of MeOH/ice mixture, the reaction mixture was quenched. The mixture then was filtered and was washed with distilled water under stirrer for 4 h at 80 °C (200 mL, 2 ×), ethanol under stirrer for 6r h at 60 °C (200 mL, 4 ×), chloroform under stirrer for 6r h at 60 °C (200 mL, 4 ×), and finally dichloromethane under stirrer for 6 h at 25 °C (200 mL, 2 ×). In the end, the yellow powder was transferred to the vacuum oven and dry at 100 °C. 11 g of Cop-150 was obtained (Fig. 1).

### Synthesis of NPC

NPC synthesized according reported method by Hu et al.^22^. 0.5 g of Cop-150 powder was placed in a ceramic boat. Then, they were spread evenly inside the boat and transferred in the furnace under nitrogen gas flow (5 °C min^−1^) at 800 °C and for 5 h. The obtained black powders were immersed in 20% HF under magnetic stirring for 24 h to remove aluminum species. The obtained solution was centrifuged at 9000 rpm for 10 min. This washing process was repeated 4 times. Finally, the obtained black products were washed three times with distilled water and kept in a vacuum oven at 40 °C for 24 h to dry (Fig. 1).

### Batch adsorption experiments

The batch adsorption method was used to investigate the removal of phenol by Cop-150 and NPC, study the equilibrium isotherms, kinetics, and thermodynamics. According to the conditions of 29 runs proposed by Box–Behnken response surface methodology, concentration of initial phenol, pH, contact time and temperature variables were examined. A shaker incubator was used to adsorption experiments at identified temperature and 175 rpm. The adsorption amount of the phenol was measurement by UV–Vis instrument at 269 nm.

Phenol removal percentage (R_e_ (%)) and equilibrium adsorption capacity (q_e_ (mg g^−1^)) were calculated under different experimental conditions including initial phenol concentration, pH, contact time and temperature. The R_e_% and q_e_ of phenol is calculated using Eqs. (1) and (2), respectively:

1 $$\mathrm{Re\%}=\frac{{\mathrm{C}}_{0}-{\mathrm{C}}_{\mathrm{e}}}{{\mathrm{C}}_{0}}\times 100$$

2 $${\mathrm{q}}_{\mathrm{e}}= \frac{({\mathrm{C}}_{0}-{\mathrm{C}}_{\mathrm{e}})\mathrm{V}}{\mathrm{m}}$$

where C_0_ (mg g^−1^), C_e_ (mg g^−1^), V (l), and m (g) are the initial and equilibrium concentrations of phenol, volume of the solution, and the adsorbent, respectively^27^.

## Results and discussion

**Figure 2:**
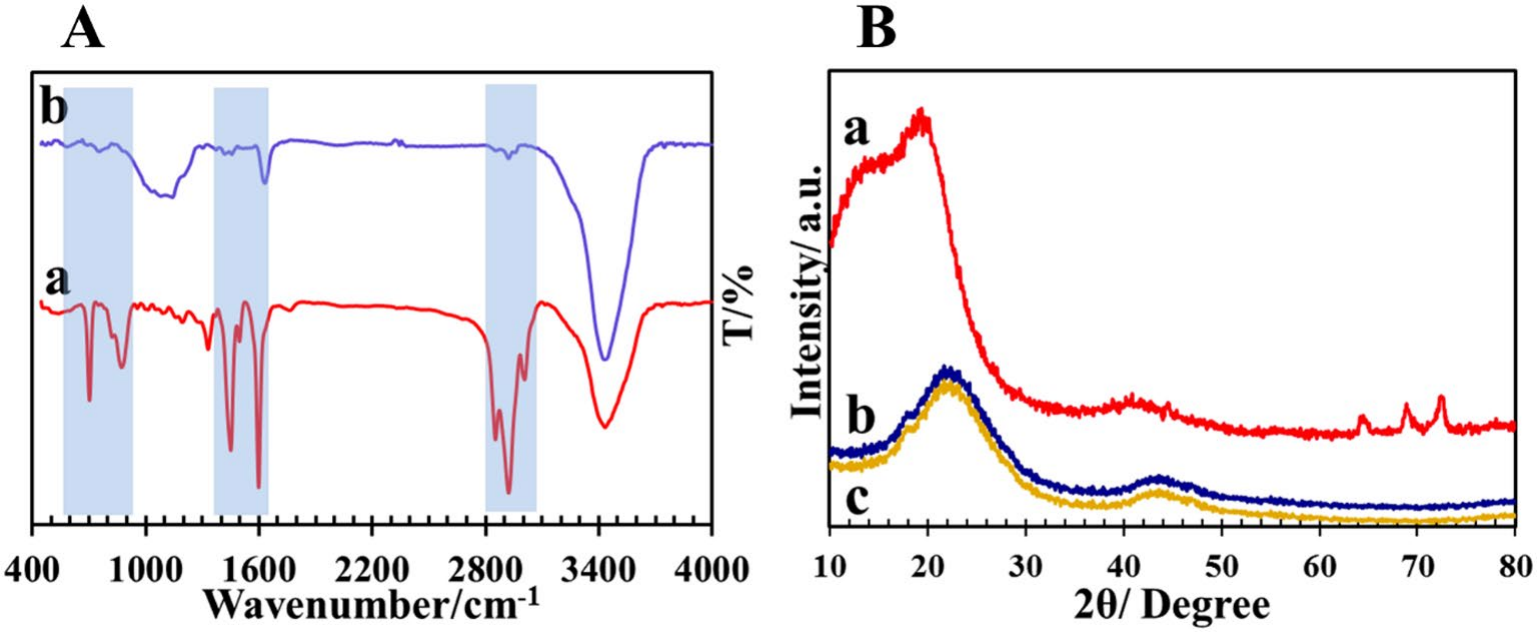
(**A**) FT-IR spectrums of (a) Cop-150, (b) NPC; (**B**) XRD pattern of (a) Cop-150, (b) NPC, (c) NPC after adsorption process.

To study functional groups of Cop-150 and NPC nanoparticles were used FT-IR analysis (Fig. 2A) and Table 2. According to the results, the peaks of the NPC spectrum than the Cop-150 spectrum are weaker (1633 cm^−1^ and 3431 cm^−1^) and/or have been removed (600–900 cm^−1^, 1000–1200 cm^−1^, 1400–1600 cm^−1^, and 2800–3000 cm^−1^), which related to the calcination process and washing with HF (see Fig. 2A-b) ^28^.

**Table 2:** Results of surface chemistry analysis of adsorbents using FT-IR.

Sample	Function groups	Present work	Reference wavenumber	Ref.
Cop-150	Aromatic C–H bending	600–900	600–900	^2,26^
	Aliphatic C–H bending	1000–1200	1000–1200	
	Aromatic C–C stretching	1400–1600	1400–1600	
	C–H stretching	< 3000	< 3000	
	Aromatic C–H stretching	> 3000	> 3000	
NPC	Carboxylic groups	1633	1560	^2,28^
	Hydroxyl groups	3431	3254	

Figure 2B is displayed the XRD pattern of (a) Cop-150 and )b) NPC. The plane 002 and 100 corresponds to the broad peaks at 2θ = 25° and 44°, which refers to graphitic carbon^2,29^. The presence of several small peaks in Fig. 2B-a indicates the existence of alumina in the Cop-150 structure. As a result of the dehydration reaction of Cop-150 aluminum hydroxide components, alumina is formed, which was completely removed due to HF treatment in the NPC structure Fig. 2B-b. Furthermore, according to the XRD of the NPC after the adsorption process, no specific changes were observed, that confirms the stability of the sample (Fig. 2B-c).

**Figure 3:**
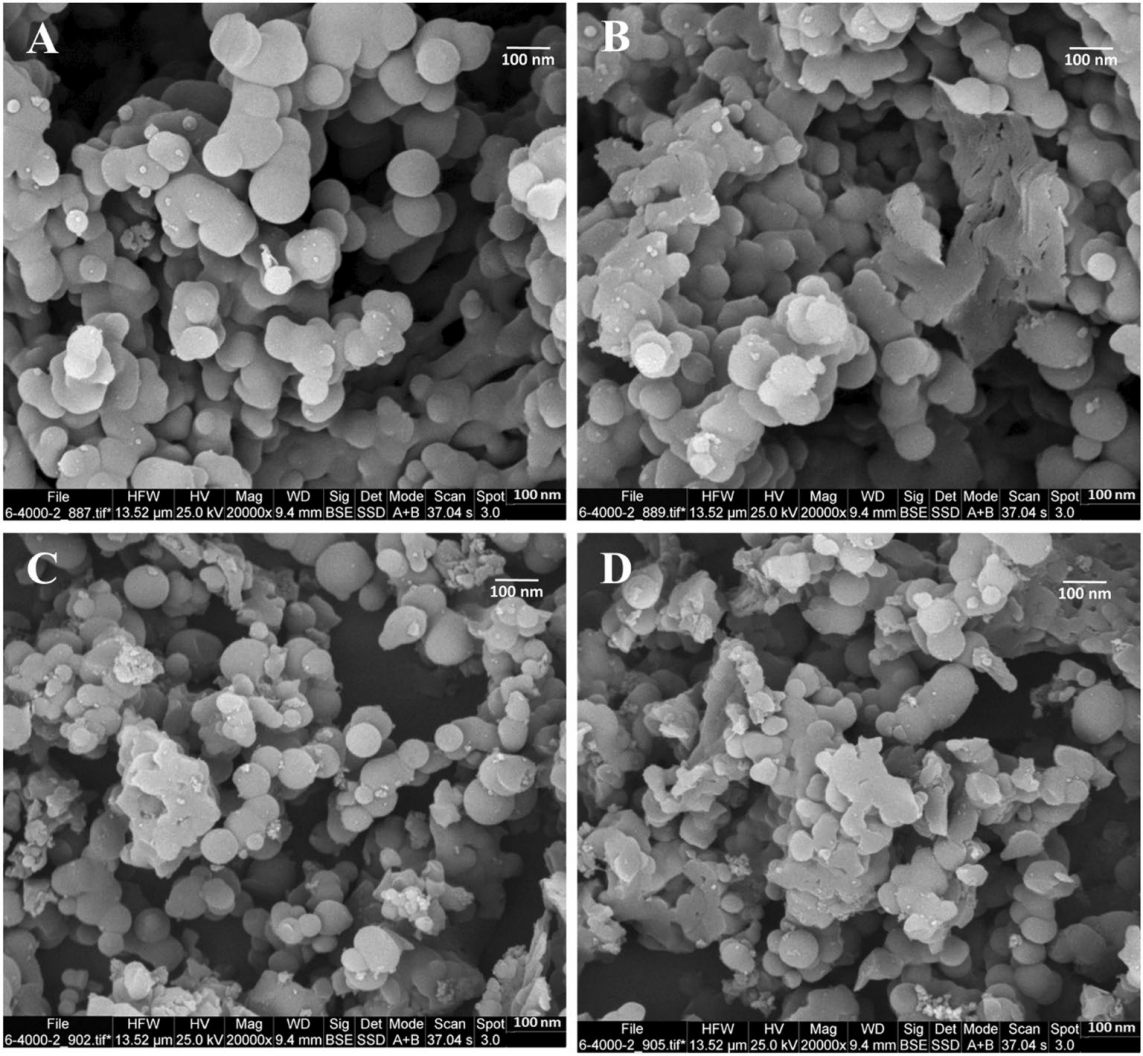
SEM images of Cop-150: (**A**) Before adsorption, (**B**) After adsorption; SEM images of NPC: (**C**) Before adsorption, (**D**) After adsorption.

The scanning electron microscope (SEM) images provide information on the surface morphology, structure, and particle size distribution of the samples. Figure 3A and C display the SEM of the Cop-150 and NPC, respectively. As shown in figures, Cop-150 and NPC have a spherical structure^2^. In addition, after the adsorption process, the spherical structure of the adsorbents has been preserved, which indicates the stability of these adsorbents (see Fig. 3B,D).

**Figure 4:**
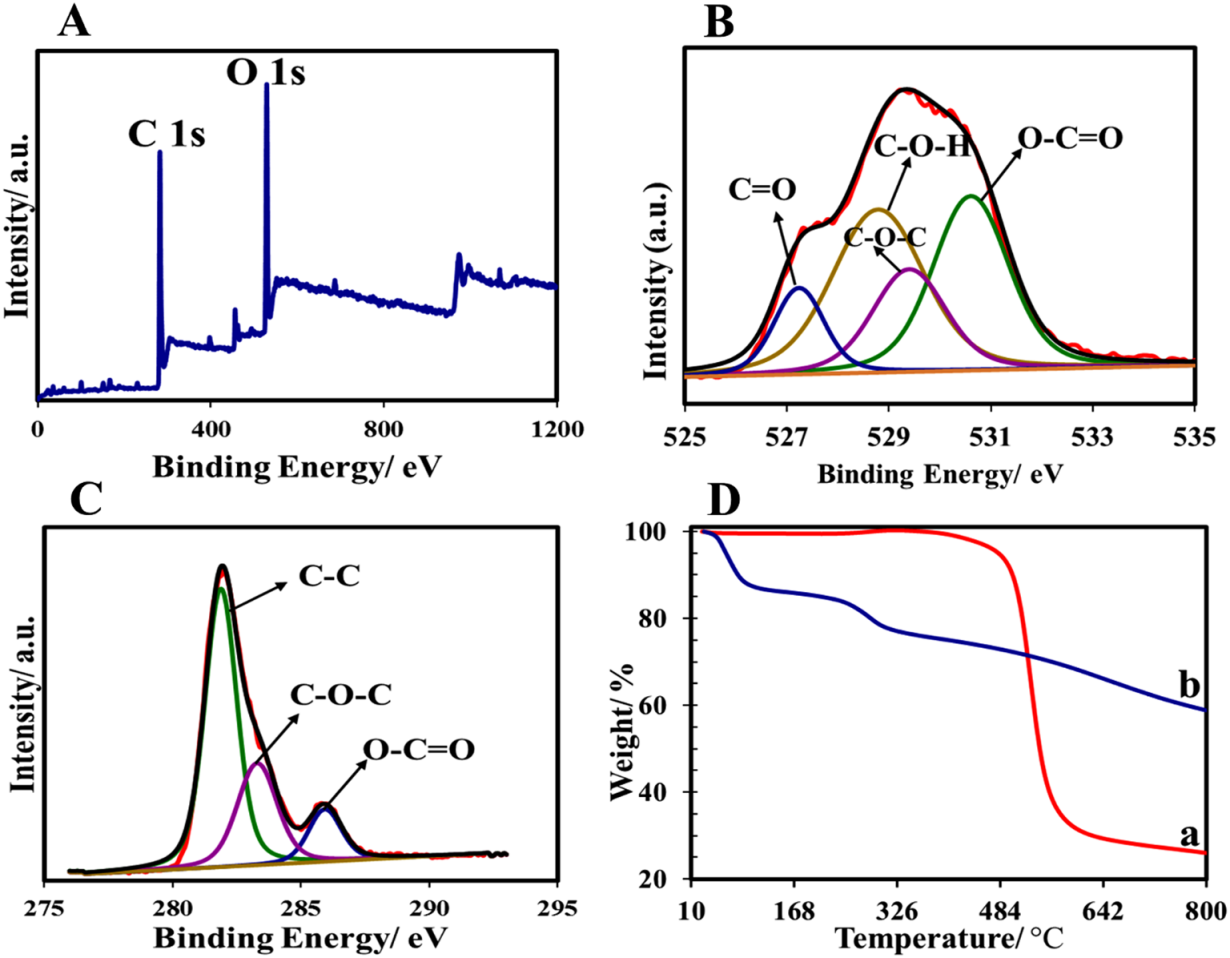
XPS spectra of NPC; (**A**) full survey spectrum, (**B**) O 1*s*, (**C**) C 1*s*; (**D**) TGA curves of (a) Cop-150, (b) NPC.

According to the XPS survey spectra (Fig. 4A), there were two major peaks at 281.9, and 527 eV corresponding to C 1*s*, and O 1*s*. Table 3 lists the C, and O content of the NPC. The high-resolution O 1*s* spectrum (Fig. 4B) was fitted by four peaks suggesting the existence of four oxygen species on the surface of the sample; C=O (10.9%), C–O–H (37.4%), C–O–C (18.7%), and O–C=O (33%). Also, the high-resolution C 1*s* spectrum (Fig. 4C) confirmed the presence of three different groups of carbon including C–C (60.5%), C–O–C (27.6%), and O–C=O (11.9%).

**Table 3:** The elemental concentration of surface functional groups of the NPC, determined by XPS method.

Compound	Peak position (eV)	Concentration (%)
O 1*s*		
C=O	527	10.9
C–O–H	528.8	37.4
C–O–C	529.4	18.7
O–C=O	530.6	33
C 1*s*		
C–C	281.9	60.5
C–O–C	283.3	27.6
O–C=O	285.9	11.9

Thermogravimetric analysis (TGA) was used to investigate the thermal stability of Cop-150 and NPC synthesized. Both adsorbents showed excellent thermal stability (Fig. 4D). The weight loss of Cop-150 occurred at about 445 °C, which is related to the decomposition of Al^3+^ (Fig. 4D-a)^30^. In the NPC sample, the first weight loss in (Fig. 4D-b) was observed at a temperature lower than 100 °C, which is related to the evaporation of water remains^31^. The second weight reduction occurred at a temperature of about 230 °C, which is due to the decomposition of volatile substances, and the removal of oxygen functional groups from the surface^2^.

Sample porosity information such as total pore volume, surface area, pore diameter, and Barrett–Joyner–Halenda (BJH) of the Cop-150 and NPC are listed in Table 3. The surface area values for the Cop-150, and NPC were achieved 10.84, 416.546 m^2^ g^−1^, respectively. Based on the IUPAC standard, particle sizes are divided into the following three categories^32^: Microspores with pore size < 2 nm, Mesoporous with pore size between 2 and 50 nm and Macrospores with pore size > 50.

**Figure 5:**
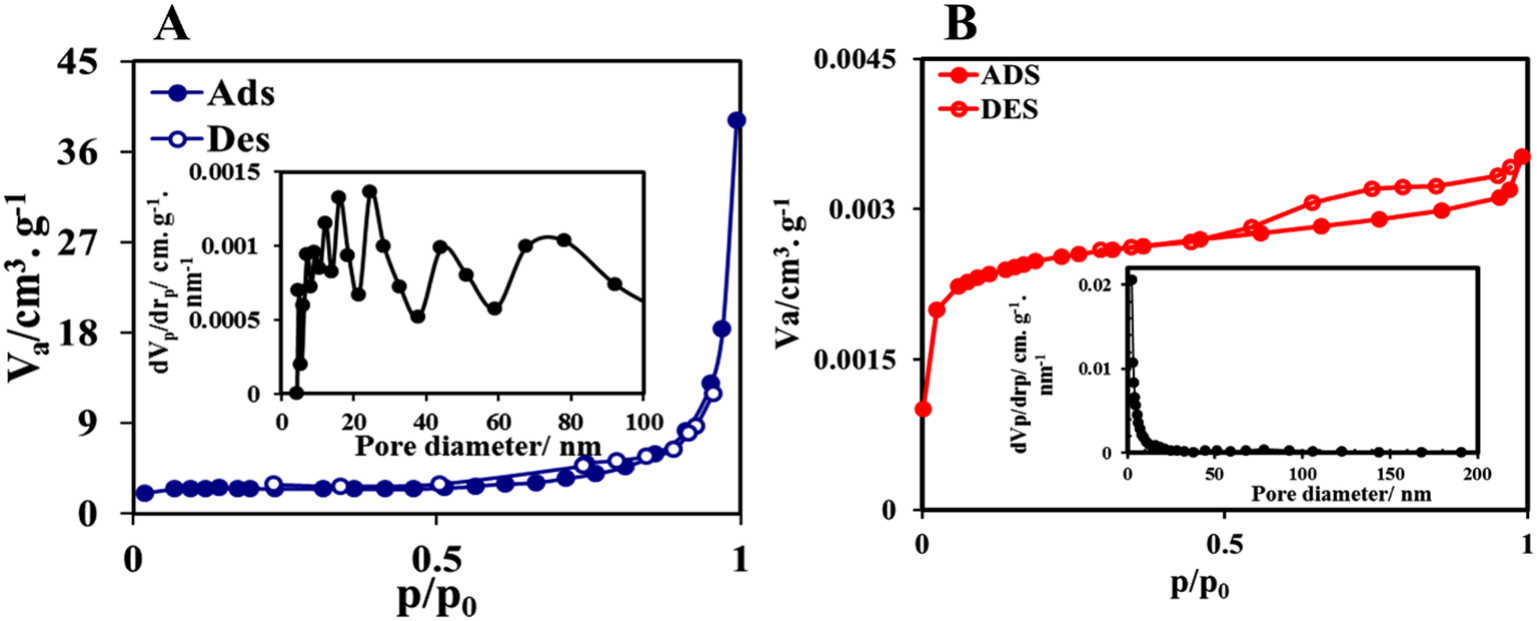
Ads/des isotherm and BJH- plot of the (**A**) Cop-150 and (**B**) NPC.

Given that the pore size diameters of Cop-150 and NPC adsorbents are between 2 and 50 nm; it can be said that they are mesoporous. The adsorption and desorption isotherms of N_2_ adsorbent were studied to evaluate the adsorption efficiency. The adsorption efficiency is a function of more surface active sites, large surface area, significant pore volume. Figure 5A shows the adsorption and desorption isotherms of adsorbent N_2_ corresponding to Cop-150, which indicated the type III isotherm (according to the IUPAC standard), which confirms the monolayer adsorption, very weak adsorbent-adsorbate interaction, and the non-porous structure of the adsorbent. Also, the isotherm of Fig. 5B corresponds to a reversible isotherm of type II with a hysteresis ring of type H_4_, which confirms the micro/ mesoporous structure^33^ The BJH plots for Cop-150 and NPC particle distributions are in the range of 2–50 nm, which confirms the particles are mesoporous (Fig. 5A,B). The results of the BJH plots are in agreement with the results of Table 4.

**Table 4:** Porous structure parameters of Cop-150 and NPC.

Material	S_BET_ (m^2^ g^−1^)	V_t_ (cm^3^ g^−1^)	Pore volume^BJH^	Average pore diameter (nm)
Cop-150	10.84	22.831	0.054	0.057
NPC	416.546	2.4	0.049	0.121

## BBRSM

Response surface method (RSM) is a way to evaluate the communication between adjustable experimental parameters and obtain results. This method, which is a multivariate statistical method, has attracted much attention as one of the best design software programs. The three steps of optimization of this method are: (1) performing statistically designed experiments, (2) estimating coefficients in a mathematical model, and (3) predicting the response and evaluating the adequacy of the model^34^. Box–Behnken design (BBD) has various applications, which among these can be referred to Spectro analytical method^35^, chromatographic methods, methods^36^, spectrum analysis method^37^, chromatographic^36^, electroanalysis methods^38^, and adsorption process^39–42^. In this research, performance randomization, experimental design, investigation of the main effects of operational parameters on phenol removal, and obtaining a set of variables with the highest phenol removal efficiency, the BBD method is used Design Expert 11.0.3.0. Data randomization is the defensible and unambiguous method and states that the conditions of the runs are independent of each other^43^. The response can be related to selected variables using quadratic models to the optimization of parameters. A quadratic equation model is giving by Eq. (3)^34,44^.

3 $$\mathrm{A}(\mathrm{Re})={\mathrm{B}}_{0}+\sum_{\mathrm{i}=1}^{\mathrm{k}}{\mathrm{B}}_{\mathrm{i}}{\mathrm{X}}_{\mathrm{i}}+\sum_{\mathrm{i}=1}^{\mathrm{k}}{\mathrm{B}}_{\mathrm{ii}}{\mathrm{X}}_{\mathrm{i}}^{2}+\sum_{\mathrm{i}=1}^{\mathrm{k}}\sum_{\mathrm{j}=1}^{\mathrm{k}}{\mathrm{B}}_{\mathrm{ij}}{\mathrm{X}}_{\mathrm{i}}{\mathrm{X}}_{\mathrm{j}}+{\mathrm{e}}_{0}$$

In the above relation, response variable, constant-coefficient, linear coefficient, quadratic coefficient, cross-product coefficient (interaction coefficient) are indicated by A(Re), B_0_, B_i_, B_ii_, and B_ij_ parameters, respectively. Also, Xi and Xj are the coded variables that to achieve these variables can be used the multiple regression analysis according to Eq. (4)^45^.

4 $${\mathrm{X}}_{\mathrm{i}}=\frac{{\mathrm{X}}_{\mathrm{i}}-{\mathrm{X}}_{0}}{\mathrm{\delta X}}$$

In this equation, X_i_ and X_0_ are the real value of the independent variable and the independent variable at the center point, respectively. Also, the change values of any step are shown by δX, which is between low (− 1) and high (+ 1) levels.

### Adsorption optimization of phenol by Cop-150 and NPC using BBRSM

In this study, was investigated the effect of four essential factors pH (a), contact time (b), initial concentration of phenol (c), and Temperature (d) in the adsorption process. The range of these factors is reported in Table 5. In addition, to investigate the effect of independent factors on the adsorption efficiency of phenols by adsorbents Cop-150 and NPC, 29 runs of experimental were designed by BBRSM, which are reported in Table 6 (see raw data in Table S1). Also, to ensure the repeatability of the experiments and to prove the normal dispersion of the experimental data, the central point parameters was repeated five times.

**Table 5:** Independent variables and levels of the process for BBRSM.

Independent variables	Symbol	Levels of independent variables		
		− 1	0	1
Concentration (mg L^−1^)	a	5	27.5	50
Time (min)	b	1	20.5	40
Temperature (°C)	c	25	42.5	60
pH	d	2	6	10

**Table 6:** Independent variables and levels of the process for BBRSM.

Run	a	b	c	d	Cop-150		NPC	
					Re (%) (actual)	Re (%) (predicted)	Re (%) (actual)	Re (%) (predicted)
1	27.5	20.5	42.5	27.5	82.77	86.39	91.53	90.83
2	50	20.5	42.5	50	43.14	41.78	55.55	52.62
3	50	20.5	42.5	50	10.73	8.05	77.91	75.23
4	50	40	42.5	50	46.73	50.43	91.65	92.97
5	5	1	42.5	5	91.38	85.95	96.51	93.54
6	27.5	1	42.5	27.5	45.85	49.4	80.72	82.51
7	27.5	20.5	42.5	27.5	87.8	86.39	90.60	89.53
8	27.5	20.5	60	27.5	49.76	51.35	71.34	75.23
9	27.5	20.5	42.5	27.5	87.43	86.39	91.72	88.15
10	27.5	40	60	27.5	54.61	50.65	75.12	75.23
11	27.5	40	42.5	27.5	30.74	30.05	72.33	71.93
12	27.5	20.5	42.5	27.5	88.18	86.39	90.97	91.39
13	50	20.5	25	50	41.7	46.84	61.6	62.61
14	5	20.5	42.5	5	34.97	35.22	78.05	77.44
15	27.5	20.5	25	27.5	30.93	27.61	48.46	46.89
16	50	1	42.5	50	31.24	27.02	52.37	55.11
17	50	20.5	60	50	28.68	28.09	82.22	82.86
18	5	20.5	60	5	92.41	91.43	96.51	94.31
19	5	20.5	25	5	26.77	31.52	79.08	82.66
20	27.5	1	25	27.5	40.07	41.59	77.36	75.21
21	27.5	1	42.5	27.5	39.88	44.73	93.77	95.77
22	27.5	20.5	60	27.5	45.1	49.31	82.77	81.93
23	5	40	42.5	5	37.03	39.52	65.74	68.83
24	27.5	40	25	27.5	47.71	45.55	84.26	84.91
25	27.5	20.5	42.5	27.5	85.75	86.39	91.72	89.52
26	27.5	1	60	27.5	77.92	77.64	93.34	96.54
27	5	20.5	42.5	5	63.69	62.62	65.74	63.27
28	27.5	40	42.5	27.5	40.44	41.05	74.38	75.23
29	27.5	20.5	25	27.5	37.83	31.89	42.68	43.81

### Analysis of variant (ANOVA)

**Figure 6:**
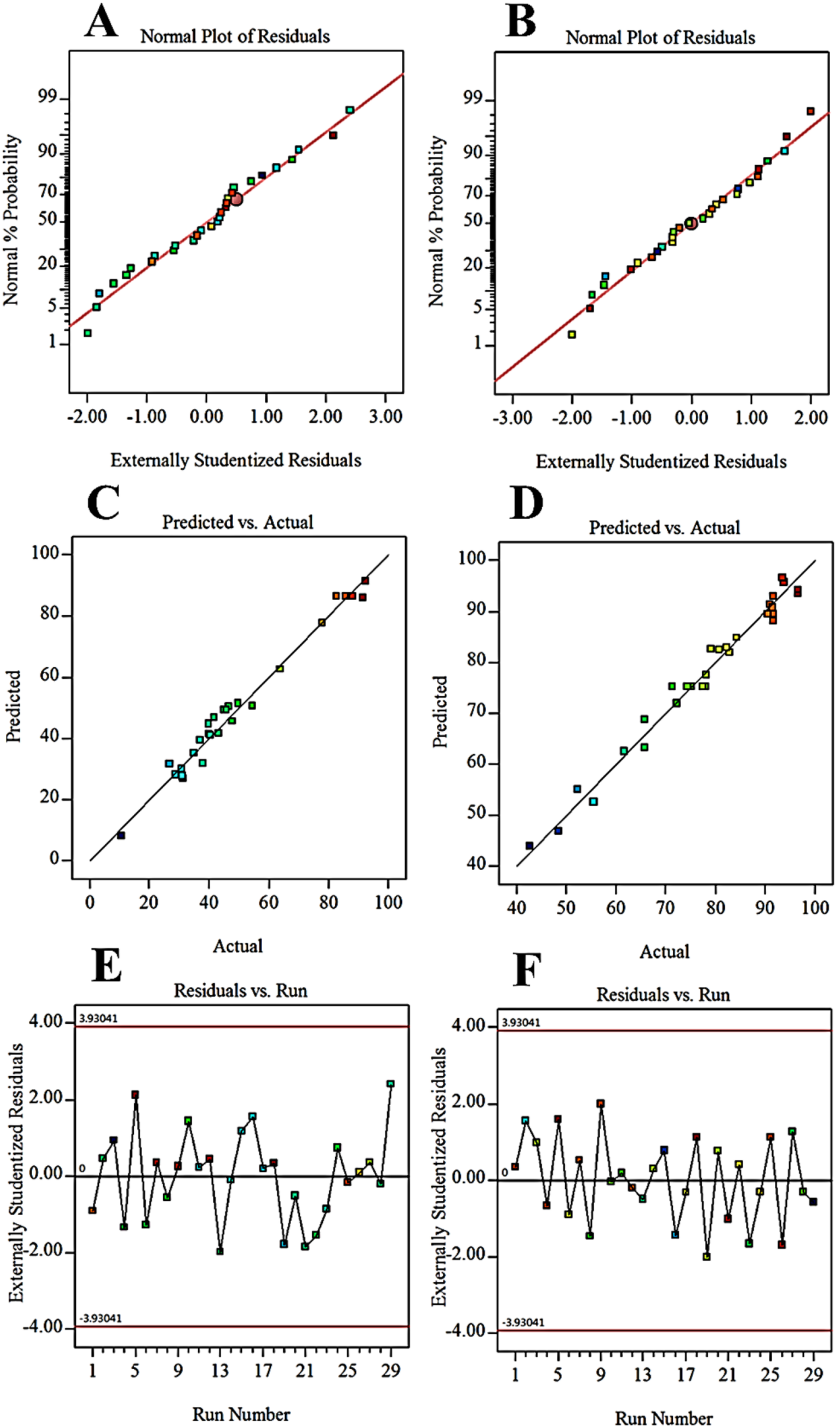
The curve of (a) the normal probability, and (b) the predicted response versus actual response.

To analyze the data obtained from the adsorption of phenol by synthesized adsorbents, four linear, 2FI, quadratic and cubic models were examined, which the quadratic model was recognized as the best model. R^2^ is a very effective parameter in the study of experimental responses. A value of R^2^ > 0.97 for Cop-150 and NPC indicates that the model is highly accurate^46^. Moreover, is observed a significant and very suitable correlation between the predicted and adjusted R^2^ (i.e. 0.96 for Cop-150). Also, for NPC, predicted R^2^ and adjusted R^2^ were equal to 0.90 and 0.95, respectively. On the other hand, the correlation between actual values and predicted values is well seen in Fig. 6A and B, which confirms the results obtained from Table 7.

**Table 7:** Model summary statistics for phenol adsorption response by Cop-150 and NPC.

Source	Sequential p-value	Adjusted R^2^	Predicted R^2^
Cop-150			
Linear	0.1851	0.09	0.04
2FI	0.2436	0.186	0.094
Quadratic	**0.0001**	**0.965**	**0.904**
Cubic	0.192	0.978	0.514
NPC			
Linear	0.83	0.099	0.471
2FI	0.0029	0.46	0.123
Quadratic	** < 0.0001**	**0.96**	**0.90**
Cubic	0.121	0.98	0.97

Significant values are in bold.

Note, the difference between predicted R^2^ and adjusted R^2^ should be about 0.2 or less for the model to be significant^47,48^. In this research work, this difference is minimal for both adsorbent samples, as a result, this the model has high accuracy (Table 7).

The P-value is indicated the importance of each coefficient. The F-values is shown the strength of the interaction between each independent variable. For model parameters to be significant, the p-value must be < 0.05, and the F value must be > 1^44^. Table 8 shows the analysis of Variant (ANOVA) to the adsorption of phenols by the proposed adsorbents. The large F-value and the low P-value confirm the significance of the model for the adsorption of phenol by Cop-150 and NPC, as the Table shows. The Lack of fit for the models was 0.064 and 0.37 for Cop-150 and NPC, respectively. These data confirm the non-significance of the Lack of Fit than the pure error. Also, the effect of each factor, regression coefficients, standard effect values, and standard errors are reported in Table 9. Equations (5) and (6) show the coded equation of the ANOVA results for the adsorption of phenol by Cop-150 and NPC, respectively.

5 $$ \begin{aligned} {\text{Q}}_{{1}} = & { 86}.{39} - {12}*{\text{a}} - {5}.{76}*{\text{b}} + { 1}0.{29}*{\text{c}} + { 1}.{58}*{\text{d}} + { 17}.{\text{46a}}*{\text{b}} - { 19}.{\text{67 a}}*{\text{c}} - {15}.{\text{28 a}}*{\text{d}} \\ & - {7}.{\text{74 b}}*{\text{c}} - { 2}0.0 \, *{\text{a}}^{{2}} - {15}.{63}*{\text{b}}^{{2}} - { 16}.{9}0 \, *{\text{c}}^{{2}} - { 29}.{45 }*{\text{d}}^{{2}} \\ \end{aligned} $$

6 $$ \begin{aligned} {\text{Q}}_{{2}} = & { 71}.{36} + {5}.0{5}*{\text{a}} + {1}.{93}*{\text{b}} - 0.0{9}*{\text{c}} - 0.{39}*{\text{d}} - {1}.{\text{42a}}*{\text{d}} + {12}.{\text{22 b}}*{\text{c}} \\ & - {2}0.{\text{93 b}}*{\text{d}} - {14}.{\text{27 c}}*{\text{d}} + {13}.{48 }*{\text{a}}^{{2}} + {2}.0{8}*{\text{d}}^{{2}} \\ \end{aligned} $$

**Table 8:** Analysis of variance for the modified quadratic.

Source	Sum of squares	df	Mean square	F-value	p-value	
Cop-150						
Model	15,259.41	12	1271.61	60.50	< 0.0001	Significant
a	1729	1	1729	82.27	0.0001	
b	397.7	1	397.7	18.92	0.0001	
c	1270.57	1	1270.57	60.47	< 0.0005	
d	30.037	1	30.03	1.43	< 0.24	
ab	1219.62	1	1219.62	58.03	< 0.0001	
ac	1547.11	1	1547.11	73.61	0.0024	
ad	934.16	1	934.16	44.45	< 0.0001	
bc	239.56	1	239.57	11.4	0.0038	
a^2^	2600.32	1	2600.32	123.73	< 0.0001	
b^2^	1585.05	1	1585.05	75.42	< 0.0001	
c^2^	1851.62	1	1851.62	88.1	< 0.0001	
d^2^	5625.4	1	5625.4	267.67	< 0.0001	
Residual	336.26	16	21.02			
Lack of fit	316.47	12	26.37	5.33	0.0597	Not significant
Pure error	19.79	4	4.95			
Cor total	15,595.67	28				
NPC						
Model	6014.56	10	601.46	40.76	< 0.0001	Significant
a	306.13	1	306.13	20.75	0.0002	
b	44.51	1	44.51	3.02	0.0995	
c	8.78	1	8.78	0.5952	0.4504	
d	1.80	1	1.80	0.1218	0.7312	
ad	433.96	1	433.96	29.41	< 0.0001	
bc	668.17	1	668.17	45.29	< 0.0001	
bd	1752.66	1	1752.66	118.79	< 0.0001	
cd	814.15	1	814.15	55.18	< 0.0001	
a^2^	953.65	1	953.65	64.63	< 0.0001	
d^2^	759.72	1	759.72	51.49	< 0.0001	
Residual	265.58	18	14.75			
Lack of fit	238.41	14	17.03	2.51	0.1937	Not significant
Pure error	27.17	4	6.79			
Cor total	6280.14	28

**Table 9:** The ANOVA results of the response surface modified quadratic model.

Cop-150			
Std. dev	4.58	R^2^	0.98
Mean	52.46	Adjusted R^2^	0.9623
C.V. %	8.74	Predicted R^2^	0.9185
PRESS	1271.01	Adeq Precision	27.1654
− 2 Log likelihood	153.37	BIC	197.14
		AICc	203.63

NPC			
Std. dev	3.84	R^2^	0.96
Mean	77.80	Adjusted R^2^	0.9342
C.V. %	4.94	Predicted R^2^	0.8903
PRESS	689.15	Adeq Precision	21.8516
− 2 Log likelihood	146.52	BIC	183.56
		AICc	184.05

### Diagnostic model

Investigating the normality of the data is another way of verifying the accuracy data of the proposed model. Figure 6 shows the results of normal values and actual statistics of the proposed model for adsorbents Cop-150 and NPC. According to the results, the data obtained from the adsorption by Cop-150 (Fig. 6A) and NPC (Fig. 6B) are close to the straight line. Therefore, it confirms the normality of the data distribution. Figure 6C and D contain the results of the experimental and the mathematical model for phenol adsorption process by Cop-150 and NPC, respectively, which indicate the reliability of the proposed model. It’s clearly that the analysis of residuals is important tool for predicting of proposed model. it also indicates the difference between real value and moderate value. As be shown in Fig. 6E and F, the equal distribution of residuals in adsorbed amount implied to acceptable proposal model.

### Response surface analysis

**Figure 7:**
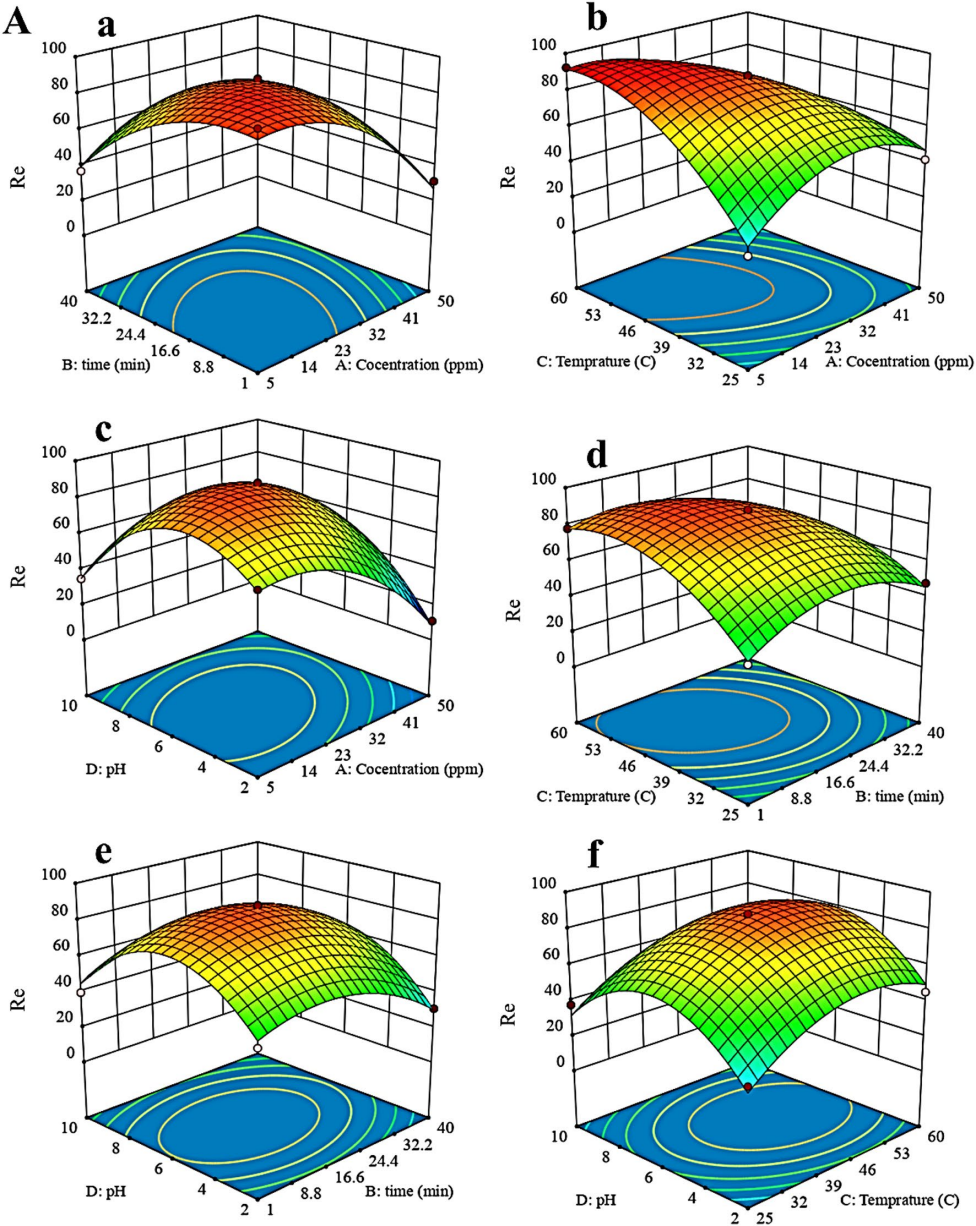

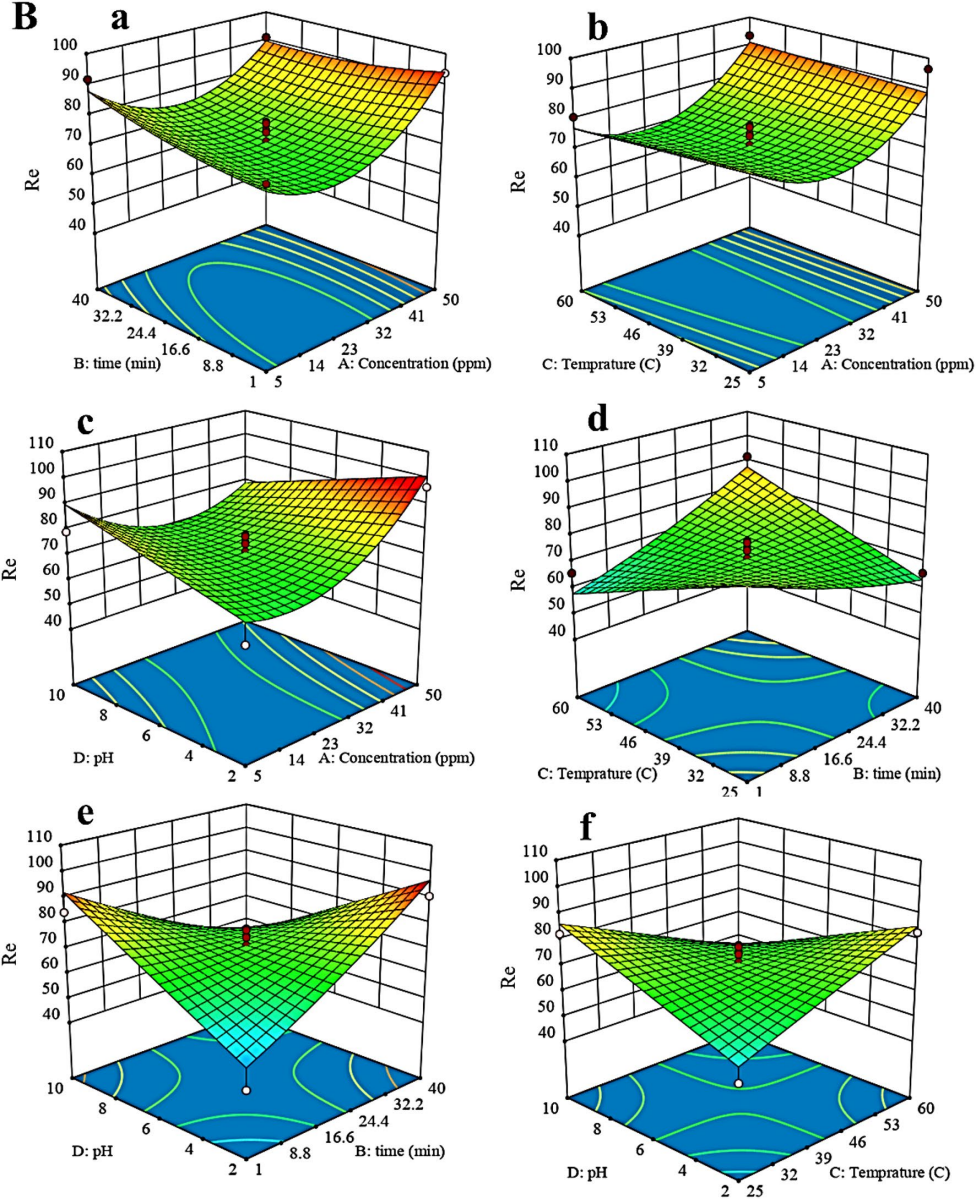
3D diagram of Re for phenol removal by (**A**) Cop-150 and (**B**) NPC.

The three-dimensional (3D) response surface plots, were used to discover the relationship between the variables (pH, time, initial concentration of phenol, and temperature). Figure 7 shows the 3D diagram of Re for phenol removal by Cop-150 and NPC according to pH, time, initial concentration of phenol, and temperature in the modified quadratic model.

### Response surface analysis to Cop-150 and NPC

As Fig. 7A-a and B-a shows, with increasing contact time and initial concentration of phenol, Re increases, which indicates these two parameters have a positive correlation with each other. However, Re was negatively correlated when the initial concentration of phenol increased beyond about 7 mg L^−1^ and contact time higher 19 min (see Fig. 7A-a). But, the increase in removal efficiency is observed with increasing the initial concentration of phenol (50 mg L^−1^) and at an equilibrium time of 15 min by NPC. The considerable adsorption efficiency in minimum time is due to the high surface area of the NPC (Fig. 7B-a).

Also, according to Fig. 7A-b and d, the temperature has a positive correlation with an initial concentration of phenol and contact time, so that with decreasing temperature to 56 °C, concentration (7 mg L^−1^) and contact time (19 min), Re has increased. Effects pH and initial concentration of phenol are shown in Fig. 7A-c and B-c. Adsorption by Cop-150 and NPC occurs in pH 4.6 and 7, respectively. PZC of Cop-150 is equal to 2.5 and to NPC is 5.4 (see raw data in Table S2). At pH < PZC, the adsorbents surface is positive, and since phenol has a positive charge at acidic pHs, the electrostatic repulsion between these charges and the sorbents surface load reduces the amount of adsorption^27^. At pHs > PZC, the Cop-150 and NPC charge is negative, and the electrostatic attraction between it and the positive charge of phenol increases the adsorption rate. According to Fig. 7B-e and f, pH is positive interaction with temperature as well as with contact time. After reaching the equilibrium point, they have a negative effect (Fig. 1).

The Fig. 7B-b confirm that with increasing temperature, the adsorption efficiency of phenol by NPC is increased. which indicates the adsorption process by NPC is endothermic^31^. Also, the strong interaction between phenol molecule and the NPC molecules is due to the increase in temperatures with increased adsorption.

At low temperatures, the equilibrium adsorption decreases with increasing adsorption time. But at high temperatures, this trend is reversed (i.e. over time an equilibrium adsorption increases). At medium temperatures of about 28 °C, the equilibrium adsorption rate is independent of time (Fig. 7B-d). As can be seen from Fig. 7B-e and f, as the contact time and temperature rise from low too high in acidic pHs, the Re increases as well. This behavior continues until it reaches equilibrium (i.e. contact time 15 min and temperature about 28 °C. But in basic pHs, this trend is reversed. According to the saddle nature of the response surface displayed, at neutral pH the rate of adsorption is time-independent. Therefore, according to the 3D diagrams presented for Cop-150 and NPC can be inferring follow results.

For the adsorption of phenol by Cop-150, temperature than concentration, pH, and contact time have the greatest effect on the adsorption efficiency, which can be well seen from the graphs. To NPC, the increasing trend of concentration is much higher than pH, temperature, and contact time. Also as stated in the ANOVA table can be assumed that the effect of the initial concentration on the final adsorption rate was very significant.

### Optimization and validation

After fitting the model, BBRSM optimization was used to optimize the selected independent parameters. According to the BBRSM predict, the value of Re is equal to 94.585 mg g^−1^ to Cop-150 under the condition of the pH 4.638, the contact time = 19.695 min, the temperature = 56.8 °C, and the initial concentration of phenol = 6.902 mg L^−1^ with desirability equal to 1.0. Similarly, the Re value for the NPC is equal to 96.70 mg g^−1^ in a condition of pH 7.042, the contact time = 15.738 min, temperature = 28.3 °C, and the initial concentration of phenol = 49.252 mg L^−1^ with desirability equal to 1.0.

## Isotherm investigations of adsorption of phenol

In this research work, different models of adsorption equilibrium isotherms such as Langmuir^49,50^, Freundlich^7^, Temkin^6,51^, and Dubinin–Radushkovich (D–R)^52–54^ were used to analyzed the experimental data (see raw data in Table S3). Linear form this isotherm is showing in the Table 10.

**Table 10:** Linear form of adsorption isotherms.

Isotherms name	Linear form	Parameters	Ref.
Langmuir	$\frac{{\mathrm{c}}_{\mathrm{e}}}{{\mathrm{q}}_{\mathrm{e}}}=\frac{1}{{\mathrm{K}}_{\mathrm{L}}{\mathrm{q}}_{\mathrm{m}}}+\frac{{\mathrm{c}}_{\mathrm{e}}}{{\mathrm{q}}_{\mathrm{m}}}$	q_m_ (mg g^−1^) = Maximum adsorption capacity K_L_ (L mg^−1^) = Langmuir adsorption constant	^7,55^
Freundlich	${\mathrm{Logq}}_{\mathrm{e}}={\mathrm{LogK}}_{\mathrm{F}}+\frac{1}{\mathrm{n}}{\mathrm{LogC}}_{\mathrm{e}}$	n = The intensity adsorption K_F_ = The adsorption capacity	^7,55^
Tamkin	${\mathrm{q}}_{\mathrm{e}}= {\mathrm{B}}_{1}{\mathrm{lnK}}_{\mathrm{T}}+{\mathrm{B}}_{1}{\mathrm{lnC}}_{\mathrm{e}}$ B_1_ = $\frac{\mathrm{RT}}{\mathrm{b}}$(10)	B_1_ (J mol^−1^) and K_T_ (L g^−1^) = Temkin constants b = The heat of adsorption k = The maximum bond energy	^2^
D–R	${\mathrm{ln}(\mathrm{q}}_{\mathrm{e}})={\mathrm{ln}(\mathrm{q}}_{\mathrm{m}})-\upbeta {\upvarepsilon }^{2}$ $\mathrm{E}= \frac{1}{{(2\upbeta )}^\frac{1}{2}}$	β (KJ^2^ mmol^−2^) = The coefficient of mean free adsorption ε (J mmol^−1^) = Polanyi potential E (KJ mmol^−1^) = Amount of energy	^56^

The isotherms constant values are showed in Table 11. Since the R^2^ value of the Freundlich isotherm (for both adsorbents) is higher than the other three isotherms, this isotherm is most consistent with the experimental data. The adsorption of phenol molecules takes place on the surfaces of sorbents which is heterogeneous, and the adsorbed phenol molecules where interacting with each other according to the Freundlich model. The fitting degree of the isotherms is as follows:

$$ {\text{Cop}} - {15}0:{\text{ Freundlich}} > {\text{ Temkin}} > {\text{ D}} - {\text{R}} > {\text{ Langmuir}}. $$

$$ {\text{NPC}}:{\text{ Freundlich }} > {\text{ Langmuir }} > {\text{ Temkin }} > {\text{ D}} - {\text{R}}. $$

**Table 11:** Isotherm constant and correlation coefficients calculated for phenol removal by Cop-150 and NPC.

Freundlich				
Parameter	n	K_F_	R^2^	
Cop-150	0.79	6.024	0.97	
NPC	0.58	5.15	0.97	

Langmuir				
Parameter	q_m_	K_L_	R_L_	R^2^
Cop-150	50	0.10	0.66	0.99
NPC	500	0.027	0.42	0.95

Temkin				
Parameter	B_1_	K_T_	R^2^	
Cop-150	0.48	2.17	0.98	
NPC	0.25	2.49	0.93	

D–R				
Parameter	β_*10_^–3^	q_m_	E_*10_^–3^	R^2^
Cop-150	0.0007	33.8	0.0014	0.95
NPC	0.002	106.39	0.004	0.88

Also, the adsorption efficiency of amoxicillin on NPC was investigated. Based on the results reported in Table S6, the value of q_m_ equal to 344.827 was obtained.

### Study of the effect of contact time and kinetic adsorption of phenol

The kinetics models of the pseudo-first-order (PFO) (Lagergren and Svenska)^54^, pseudo-second-order (PSO) (Ho and Mckay)^57^, Elovich (Elovich and Larinov)^58^, and intraparticle diffusion to the investigating of the kinetic data were used (see raw data in Table S4). Table 12 shows the linear form of each kinetic Eqs:

**Table 12:** Linear form of kinetic models.

Eq	Linear form	Parameters	Ref.
PFO	$\mathrm{log}\left({\mathrm{q}}_{\mathrm{e}}-{\mathrm{q}}_{\mathrm{t}}\right)={\mathrm{logq}}_{\mathrm{e}}-\frac{{\mathrm{K}}_{1}.\mathrm{t}}{2.303}$	q_t_ (mg g^−1^) = Adsorption capacity at time (t) q_e_ (mg g^−1^) = Adsorption capacity at equilibrium k_1_(min^−1^) = The PFO rate constant	^57^
PSO	$\frac{\mathrm{t}}{{\mathrm{q}}_{\mathrm{t}}}=\frac{1}{{\mathrm{K}}_{2}{\mathrm{q}}_{\mathrm{e}}^{2}}+\frac{1}{{\mathrm{q}}_{\mathrm{e}}(\mathrm{t})}$	K_2_ (g mg^−1^ min) = The rate constant of the PSO	^57^
Elovich	${\mathrm{q}}_{\mathrm{t}}=\frac{1}{\upbeta }\mathrm{ln}(\mathrm{\alpha \beta })+\frac{1}{\upbeta }$ ln (t)	$\mathrm{\alpha }$= The initial phenol adsorption rate $\upbeta$ (g mg^−1^) = The surface coverage	^57^
Intraparticle diffusion	${\mathrm{q}}_{\mathrm{t}}={\mathrm{K}}_{\mathrm{dif}}{\mathrm{t}}^\frac{1}{2}+\mathrm{C}$	C (mg g^−1^) = a constant of the model	^57^

**Figure 8:**
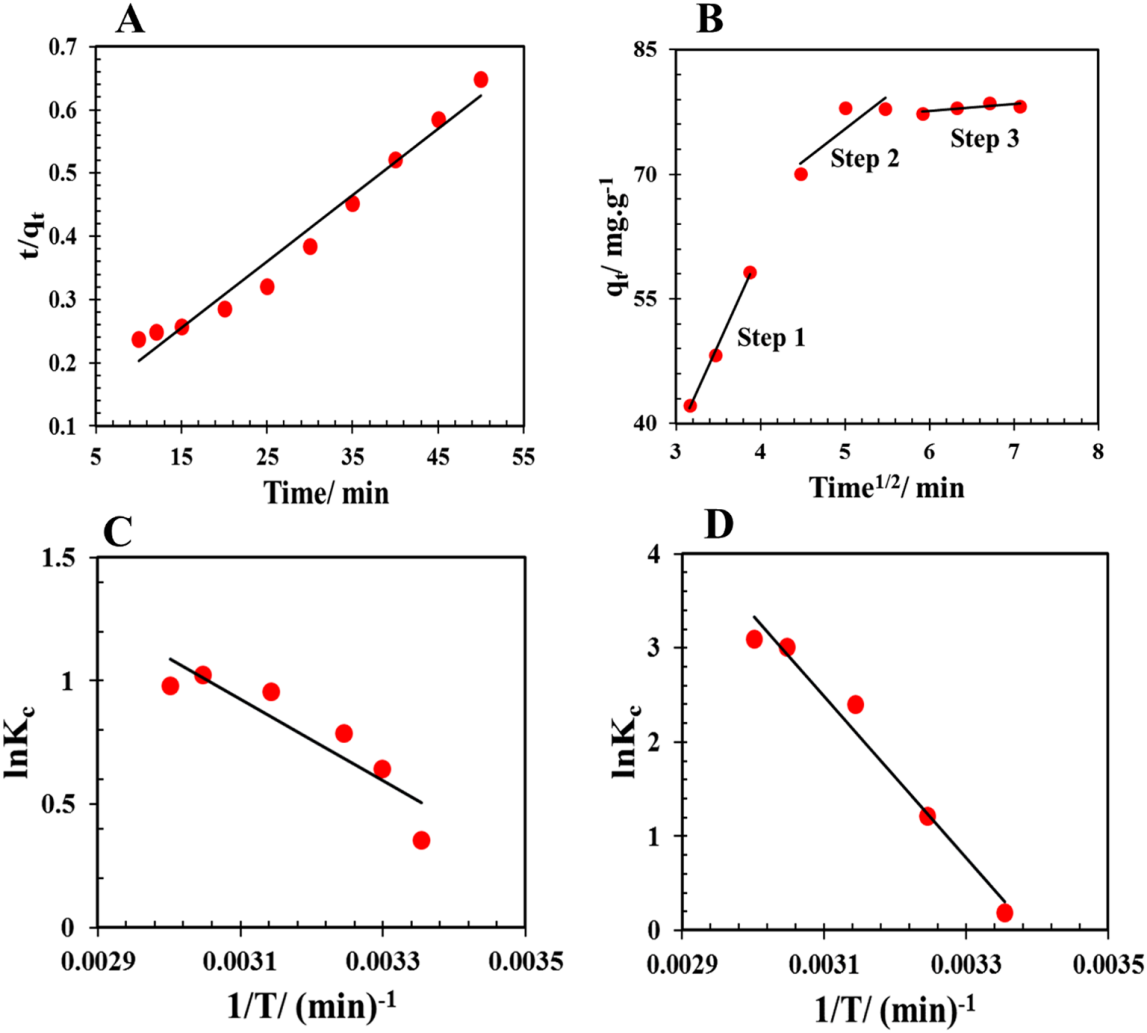
(**A**) t/q_t_ versus time graph of the NPC; (**B**) The steps of the intraparticle diffusion model for phenol removal by NPC; lnK° versus 1/T graph to the adsorption of phenol by the (**C**) Cop-150 and (**D**) NPC.

According to the results reported in Table 13, PSO kinetic model was able to well describe the experimental data obtained for the adsorption of phenol by both Cop-150 and NPC adsorbents (Fig. 8A). Figure 8A shows the good linear relation between time (t) and t/qt. Also, the possible mechanism of phenol uptake includes the following steps (Fig. 8B)^2,32^:

- Step 1: Bulk diffusion.
- Step 2: Film diffusion.
- Step 3: Pore diffusion or intraparticle diffusion and adsorption of phenol on the adsorbent surface.

**Table 13:** Adsorption kinetic parameters for phenol removal onto Cop-150 and NPC.

Pseudo-first-order				
Parameter	R^2^	K_1_ (min^−1^)	Q_e, Calc_ (mg g^−1^)	
Cop-150	0.92	0.050	1.80	
NPC	0.95	0.039	2.139	

Pseudo-second-order				
Parameter	R^2^	K_2_ (min^−1^)	Q_e, Calc_ (mg g^−1^)	
Cop-150	1	0.086	3.9	
NPC	0.97	0.011	95.23	

Elovich				
Parameter	R^2^	a (mg g^−1^ min^−1^)	B (mg g^−1^)	
Cop-150	0.85	0.84	1.7	
NPC	0.82	3.13721E+05	0.045	

Intraparticle				
Parameter		R^2^	K_dif_ (L min^−1^)	C
Cop-150	Step (1)	0.91	0.66	1
	Step (2)	0.97	0.126	3
	Step (3)	0.60	0.061	3.34
NPC	Step (1)	0.99	22.7	30
	Step (2)	0.76	7.86	36
	Step (3)	0.63	0.83	72.6

	Op-150	NPC		
Q_e, Exp_ (mg g^−1^)	3.62	70.085

## Adsorption thermodynamics

The effect of temperature on the adsorption of phenol was examined using thermodynamic studies (see raw data in Table S5). Equations (7) and (8) were used to calculate ln Kc and the changes of Gibbs free energy, respectively^59^:

7 $$\mathrm{lnK}^\circ =\frac{qe}{Ce}$$

8 $$\Delta \mathrm{G}^\circ = -\mathrm{RTlnK}^\circ $$

In this Eqs. K°, T (K) and R (8.314 J mol^−1^ K^−1^) are the equilibrium constant, temperature, and the universal gas constant, respectively (Table 14 and Fig. 8C and D). The achieved amounts of ∆G° to all used adsorbents here are negative, which is confirm the adsorption of phenol by them is spontaneous. The standard entropy changes (∆S°) and enthalpy changes (∆H°) for the adsorption process, were obtained from the slope and intercept lnK° versus 1/T graph (i.e. Van't Hoff relationship [Eq. (9)]^59,60^, respectively.

**Table 14:** Thermodynamic parameters for phenol removal onto NPC.

Adsorbent	Parameter	Temperature				
		298.15	308.15	318.15	328.15	333.15
Cop-150	K°	1.4	2.2	2.6	2.8	2.7
	ΔG°(Kj mol^−1^)	− 0.884	− 0.162	− 0.202	− 0.253	− 0.279
	ΔH° (Kj mol^−1^)	− 0.1373				
	ΔS° (Kj mol^−1^)	0.050				
NPC	K°	1.2	3.36	11.12	20.3	22.23
	ΔG° (Kj mol^−1^)	− 0.471	− 0.3108	− 0.637	− 0.821	− 0.859
	ΔH° (Kj mol^−1^)	0.242				
	ΔS° (Kj mol^−1^)	− 0.7151				

9 $$\mathrm{lnK}^\circ =\frac{\Delta \mathrm{S}^\circ }{\mathrm{R}}-\frac{\Delta \mathrm{H}^\circ }{\mathrm{RT}}$$

According to the results, the value of ∆H° to the adsorption of phenol onto Cop-150 and NPC is negative (exothermic) and positive (endothermic), respectively^61^. On the other hand, the reduction of the absolute value of ΔG° with temperature indicates a lower tendency for the adsorption of phenol on the adsorbents used at higher temperatures.

## Comparison of the q_m_ of NPC of this work with other adsorbents

In this research work, a very inexpensive adsorbent with easy and rapid synthesis was used to remove phenol. The results showed a significant amount of q_m_ for removal of phenol by NPC compared to other adsorbents in the literature (Table 15).

**Table 15:** Comparison of the q_m_ of NPC with q_m_ of the other adsorbents.

Sorbent	q_m_ (mg g^−1^)	Ref.
NPC	500	This study
GO/PPy	201.4	^62^
NiO@GNCC	300	^63^
GO-PNIPAM	10	^64^
Magnetic hydroxyethyl cellulose/ionic	336	^65^
Microalgae derived biochar	205	^66^
Rice straw biochar	50	^67^

## Investigation the reusability of the sorbents

**Figure 9:**
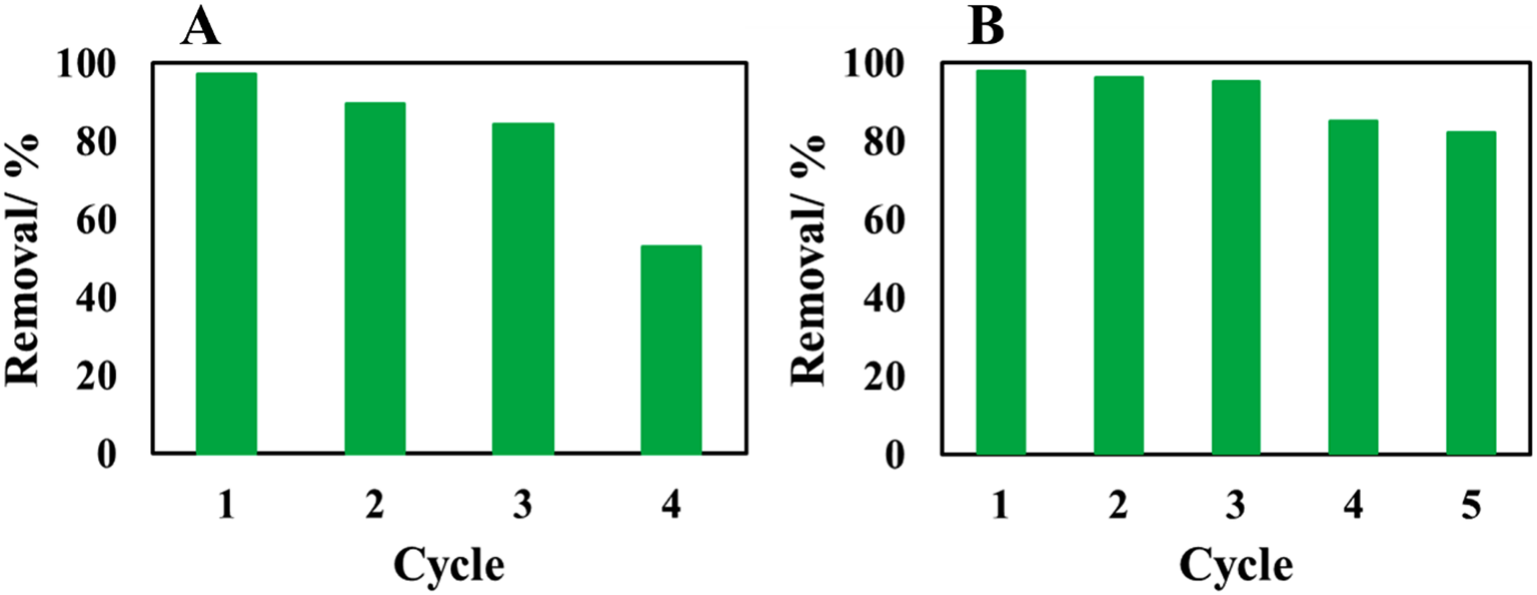
The regeneration of the (**A**) Cop-150, and (**B**) NPC.

Reusability, reproducibility and stability of adsorbents are three key factors for their widespread use. Therefore, a certain amount of adsorbent was added to 50 ml of phenol solution. After completing the adsorption process in the optimal conditions (Section “Optimization and validation”), the adsorbents were separated and washed twice with ethanol. After drying, the adsorbents were used for the next adsorption cycle. Thus, the adsorption–desorption recycling used to study of the durability of the Cop-150, and NPC sorbents (Fig. 9). As Fig. 9 shows, the Cop-150 and NPC could be used for up to four and five cycles without significantly reducing their performance, respectively. Therefore, higher, repeatability, durability and excellent stability of NPC indicate the suitability of this adsorbent to remove contaminants.

## Conclusion

The adsorption of phenol from the wastewater was studied by NPCs based on Cop-150, and NPC sorbent has an excellent q_m_ to adsorption of phenol. BBRSM was used to optimize four important factors of the pH (2–10), contact time (1–40 min), temperature (25–60 °C), and initial concentration of phenol (5–50 mg L^−1^). To analyze the data obtained from the adsorption of phenol by synthesized adsorbents, four linear, 2FI, quadratic and cubic models were examined, which the quadratic model was recognized as the best model. Excellent efficiency and high q_m_ of 500 mg g^−1^ (with a contact time of 15.738) in phenol removal were both achieved that suggest the application of NPC in water treatment. Also, more than 90% adsorption of phenol was observed at initial concentration of phenol = 49.252 mg L^−1^, contact time = 15.738 min, temperature = 28.3 °C, and pH 7.042. On the other hand, the adsorption capacity for Cop-150 in pH 4.638, the contact time = 19.695 min, the temperature = 56.8 °C, and the initial concentration of phenol = 6.902 mg L^−1^ was equal to 50 mg g^−1^. The study of adsorption isotherms displayed that the adsorption of phenol by two Cop-150 and NPC adsorbents follows the Freundlich isotherm model. Also, the kinetic data for the sorbent were fitted using the PSO model. This work indicates that proposed NPC can be considered an excellent adsorbent due to its suitable adsorption capacity, and low equilibrium time. The calculated thermodynamic adsorption parameters showed that the adsorptions of phenol onto this sorbent is spontaneous and endothermic. In addition, according to results the Cop-150 and NPC could be used for up to four and five cycles without significantly reducing their performance, respectively.

Among the limitations of phenol adsorption by NPCs, competitive adsorption can be mentioned. In real-world scenarios, the presence of other organic and inorganic compounds in wastewater can compete with phenol for adsorption sites on the NPC. This competitive adsorption can impact the overall efficiency and selectivity of phenol removal. Understanding the interactions and competition between phenol and other compounds is essential to optimize the adsorption process.

## The proposal for further research’s

Phenol is a common organic pollutant found in industrial wastewater, and its removal is crucial to ensure environmental sustainability. Adsorption using NPC materials has gained significant attention due to their high surface area, tunable pore size distribution, and excellent adsorption capacity. However, further research is needed to understand the underlying adsorption mechanism and optimize the process for efficient removal of phenol from contaminated water sources. The development of nanoporous carbons as efficient and eco-friendly nano-sorbents for phenol adsorption holds great promise for wastewater treatment applications. Researchers are actively investigating different strategies to enhance their adsorption capacity, selectivity, and stability. Additionally, efforts are being made to optimize the synthesis process and explore novel precursor materials to further improve the eco-friendliness and cost-effectiveness of these nano-sorbents. As research in this field continues to progress, nanoporous carbons have the potential to contribute significantly to the development of efficient and sustainable wastewater treatment technologies.

### Supplementary Information

Supplementary Tables.

## Data Availability

All data generated or analyzed during this study are included in this published article [and its supplementary information files].
