# Supplementary material for: Nanoporous carbons based on coordinate organic polymers as an efficient and eco-friendly nano-sorbent for adsorption of phenol from wastewater
Source: Sci Rep. 2023 Aug 12;13:13127. doi: 10.1038/s41598-023-40243-0 (PMC10423284; doi:10.1038/s41598-023-40243-0)
Supplement: Supplementary file 1 — Supplementary Tables. [file 41598_2023_40243_MOESM1_ESM.docx]

**Supporting Information’s**

**Nanoporous carbons based on coordinate organic polymers as an efficient and eco-friendly eano-sorbent for adsorption of phenol from wastewater**

**Soheila Sharafinia^1^, Alimorad Rashidi^2^*, Behnam Babaei^3^,** **[Yasin Orooji](https://www.sciencedirect.com/science/article/abs/pii/S0169433221009685#!)^4^**

^1^Department of chemistry, Faculty of Science, Shahid Chamran University of Ahvaz, Ahvaz, Iran

^2^Nanotechnology Research Center, Research Institute of Petroleum Industry (RIPI), Tehran, Iran

^3^Department of Chemistry, Faculty of Basic Science, University of Mohaghegh, Ardabili, Iran

^4^Material and Energy Research Center, Karaj, Alborz, Iran

Corresponding author.

E-mail addresses: **^2^***[rashidiam@ripi.ir](mailto:rashidiam@ripi.ir)

**Table S1.** Raw data of independent variables and levels of the process for BBRSM.

|  |  |  |  |  | **Cop-150** | | | **NPC** | | |
| --- | --- | --- | --- | --- | --- | --- | --- | --- | --- | --- |
| **Run** | **C_0_ (mg/L)** | **t (min)** | **T (ᵒC)** | **pH** | **Ads** | **Ce (mg/L)** | **Re (%)** | **Ads** | **Ce (mg/L)** | **Re (%)** |
| 1 | 27.5 | 20.5 | 42.5 | 6 | 0.099 | 4.73846 | 82.7692 | 0.052 | 2.32821 | 91.5338 |
| 2 | 50 | 20.5 | 42.5 | 10 | 0.561 | 28.4308 | 43.1385 | 0.44 | 22.2256 | 55.5487 |
| 3 | 50 | 20.5 | 42.5 | 2 | 0.877 | 44.6359 | 10.7282 | 0.222 | 11.0462 | 77.9077 |
| 4 | 50 | 40 | 42.5 | 6 | 0.526 | 26.6359 | 46.7282 | 0.088 | 4.17436 | 91.6513 |
| 5 | 5 | 1 | 42.5 | 6 | 0.015 | 0.43077 | 91.3846 | 0.01 | 0.17436 | 96.5128 |
| 6 | 27.5 | 1 | 42.5 | 2 | 0.297 | 14.8923 | 45.8462 | 0.11 | 5.30256 | 80.7179 |
| 7 | 27.5 | 20.5 | 42.5 | 6 | 0.072 | 3.35385 | 87.8042 | 0.057 | 2.58462 | 90.6014 |
| 8 | 27.5 | 20.5 | 60 | 10 | 0.276 | 13.8154 | 49.7622 | 0.16 | 7.86667 | 71.3939 |
| 9 | 27.5 | 20.5 | 42.5 | 6 | 0.074 | 3.45641 | 87.4312 | 0.051 | 2.27692 | 91.7203 |
| 10 | 27.5 | 40 | 60 | 6 | 0.25 | 12.4821 | 54.6107 | 0.14 | 6.84103 | 75.1235 |
| 11 | 27.5 | 40 | 42.5 | 2 | 0.378 | 19.0462 | 30.7413 | 0.155 | 7.61026 | 72.3263 |
| 12 | 27.5 | 20.5 | 42.5 | 6 | 0.07 | 3.25128 | 88.1772 | 0.055 | 2.48205 | 90.9744 |
| 13 | 50 | 20.5 | 25 | 6 | 0.575 | 29.1487 | 41.7026 | 0.381 | 19.2 | 61.6 |
| 14 | 5 | 20.5 | 42.5 | 10 | 0.07 | 3.25128 | 34.9744 | 0.028 | 1.09744 | 78.0513 |
| 15 | 27.5 | 20.5 | 25 | 2 | 0.377 | 18.9949 | 30.9277 | 0.283 | 14.1744 | 48.4569 |
| 16 | 50 | 1 | 42.5 | 6 | 0.677 | 34.3795 | 31.241 | 0.471 | 23.8154 | 52.3692 |
| 17 | 50 | 20.5 | 60 | 6 | 0.702 | 35.6615 | 28.6769 | 0.18 | 8.89231 | 82.2154 |
| 18 | 5 | 20.5 | 60 | 6 | 0.014 | 0.37949 | 92.4103 | 0.01 | 0.17436 | 96.5128 |
| 19 | 5 | 20.5 | 25 | 6 | 0.078 | 3.66154 | 26.7692 | 0.027 | 1.04615 | 79.0769 |
| 20 | 27.5 | 1 | 25 | 6 | 0.328 | 16.4821 | 40.0653 | 0.128 | 6.22564 | 77.3613 |
| 21 | 27.5 | 1 | 42.5 | 10 | 0.329 | 16.5333 | 39.8788 | 0.04 | 1.71282 | 93.7716 |
| 22 | 27.5 | 20.5 | 60 | 2 | 0.301 | 15.0974 | 45.1002 | 0.099 | 4.73846 | 82.7692 |
| 23 | 5 | 40 | 42.5 | 6 | 0.068 | 3.14872 | 37.0256 | 0.04 | 1.71282 | 65.7436 |
| 24 | 27.5 | 40 | 25 | 6 | 0.287 | 14.3795 | 47.711 | 0.091 | 4.32821 | 84.2611 |
| 25 | 27.5 | 20.5 | 42.5 | 6 | 0.083 | 3.91795 | 85.7529 | 0.051 | 2.27692 | 91.7203 |
| 26 | 27.5 | 1 | 60 | 6 | 0.125 | 6.07179 | 77.9207 | 0.042 | 1.81538 | 93.3986 |
| 27 | 5 | 20.5 | 42.5 | 2 | 0.042 | 1.81538 | 63.6923 | 0.04 | 1.71282 | 65.7436 |
| 28 | 27.5 | 40 | 42.5 | 10 | 0.326 | 16.3795 | 40.4382 | 0.144 | 7.04615 | 74.3776 |
| 29 | 27.5 | 20.5 | 25 | 10 | 0.34 | 17.0974 | 37.8275 | 0.314 | 15.7641 | 42.676 |

**Table S2.** PZC of COP-150 and NPC.

| **COP-150** | | | **NPC** | | |
| --- | --- | --- | --- | --- | --- |
| **pH_i_** | **pH_f_** | ΔpH | **pH_i_** | **pH_f_** | ΔpH |
| 1 | 1.552 | 0.552 | 1 | 1.652 | 0.652 |
| 2.48 | 2.71 | 0.23 | 2.48 | 2.81 | 0.33 |
| 5.922 | 5.489 | -0.433 | 5.922 | 5.589 | -0.333 |
| 9.897 | 8.715 | -1.182 | 9.897 | 9.1 | -0.797 |

**Table S3.** Effect of concentration**.**

| **Cop-150** | | **NPC** | |
| --- | --- | --- | --- |
| **C_0_ (mg/L)** | **Ads** | **C_0_ (mg/L)** | **Ads** |
| 2 | 0.029 | 5 | 0.03 |
| 5 | 0.028 | 10 | 0.044 |
| 10 | 0.039 | 15 | 0.053 |
| 15 | 0.057 | 20 | 0.057 |
| 20 | 0.073 | 30 | 0.068 |
|  |  | 40 | 0.079 |
|  |  | 50 | 0.089 |
|  |  | 60 | 0.114 |
|  |  | 70 | 0.124 |
|  |  | 80 | 0.151 |
|  |  | 90 | 0.161 |

**Table S4.** Effect of time.

| **Cop-150** | | | **NPC** | | |
| --- | --- | --- | --- | --- | --- |
| **C_0_ (mg/L)** | **time (min)** | **Ads** | **C_0_ (mg/L)** | **time (min)** | **Ads** |
| 10 | 10 | 0.138 | 50 | 5 | 0.691 |
| 10 | 15 | 0.12 | 50 | 10 | 0.488 |
| 10 | 25 | 0.079 | 50 | 12 | 0.417 |
| 10 | 30 | 0.067 | 50 | 15 | 0.3 |
| 10 | 40 | 0.059 | 50 | 20 | 0.161 |
| 10 | 45 | 0.056 | 50 | 25 | 0.069 |
| 10 | 50 | 0.057 | 50 | 30 | 0.07 |
|  |  |  | 50 | 35 | 0.076 |
|  |  |  | 50 | 40 | 0.084 |
|  |  |  | 50 | 45 | 0.081 |
|  |  |  | 50 | 50 | 0.079 |

**Table S5.** Effect of temperature**.**

| **Cop-150** | | | **NPC** | | |
| --- | --- | --- | --- | --- | --- |
| **C_0_ (mg/L)** | T (ᵒC) | **Ads** | **C_0_ (mg/L)** | T (ᵒC) | **Ads** |
| 10 | 25 | 0.111 | 50 | 25 | 0.571 |
| 10 | 30 | 0.097 | 50 | 35 | 0.329 |
| 10 | 35 | 0.09 | 50 | 45 | 0.133 |
| 10 | 45 | 0.082 | 50 | 55 | 0.08 |
| 10 | 55 | 0.079 | 50 | 60 | 0.074 |
| 10 | 60 | 0.081 | 50 | 25 | 0.571 |

**Table S6.** Isotherm constant and correlation coefficients calculated for amoxicillin removal by NPC.

| Freundlich | | | |
| --- | --- | --- | --- |
| **Parameter** | n | K_F_ | R^2^ |
|  | 0.317 | 9.487 | 0.816 |
| **Langmuir** | | | |
| **Parameter** | Q_m_ | K_L_ | R^2^ |
|  | 344.827 | 0.162 | 0.970 |
